# The Impact of Cardiac Rehabilitation on Activities of Daily Life in Elderly Patients With Heart Failure

**DOI:** 10.3389/fphys.2021.785501

**Published:** 2022-01-05

**Authors:** Mara Paneroni, Simonetta Scalvini, Ugo Corrà, Marta Lovagnini, Roberto Maestri, Antonio Mazza, Rosa Raimondo, Piergiuseppe Agostoni, Maria Teresa La Rovere

**Affiliations:** ^1^Respiratory Rehabilitation, Istituti Clinici Scientifici Maugeri IRCCS, Lumezzane (Brescia), Italy; ^2^Department of Cardiac Rehabilitation, Istituti Clinici Scientifici Maugeri IRCCS, Lumezzane (Brescia), Italy; ^3^Department of Cardiac Rehabilitation, Istituti Clinici Scientifici Maugeri IRCCS, Veruno (Novara), Italy; ^4^Department of Cardiac Rehabilitation, Istituti Clinici Scientifici Maugeri IRCCS, Montescano (Pavia), Italy; ^5^Department of Bioengineering, Istituti Clinici Scientifici Maugeri IRCCS, Montescano (Pavia), Italy; ^6^Department of Cardiac Rehabilitation, Istituti Clinici Scientifici Maugeri IRCCS, Montescano (Pavia), Italy; ^7^Department of Cardiac Rehabilitation, Istituti Clinici Scientifici Maugeri IRCCS, Tradate (Varese), Italy; ^8^Centro Cardiologico Monzino, IRCCS, Milan, Italy; ^9^Department of Clinical Sciences and Community Health, Cardiovascular Section, University of Milan, Milan, Italy

**Keywords:** activities of daily living, metabolic requirement, oxygen uptake, cardiac rehabilitation, elderly, chronic heart failure

## Abstract

**Background:** In elderly chronic heart failure (HF) patients, activities of daily living (ADLs) require the use of a high proportion of patients’ peak aerobic capacity, heart rate, and ventilation.

**Objectives:** To assess the effects of short-term comprehensive cardiac rehabilitation (CR) on the metabolic requirement of ADLs in elderly patients with chronic HF.

**Methods:** The study population comprised 99 elderly chronic HF patients (mean age 72 ± 5 years, 80% male, 61% ejection fraction <40%, mean NT-proBNP 2,559 ± 4,511 pg/ml) participating in a short-term (mean days 19 ± 7) residential CR program. Before and after CR, participants, while wearing a portable ergospirometer, performed a standardized ADL battery: ADL1 (getting dressed), ADL2 (folding 8 towels), ADL3 (putting away 6 bottles), ADL4 (making a bed), ADL5 (sweeping the floor for 4 min), ADL6 (climbing 1 flight of stairs carrying a 1.5 Kg load), and ADL7 (a standard 6-min walking test).

**Results:** After CR, task-related oxygen uptake did not change in any of the domestic ADLs. Notably, there was a significant decrease in the cumulative time required to perform ADLs (ADL 1–4 and ADL6; from 412 ± 147 to 388 ± 141 s, *p* = 0.001) and a reduction in maximal heart rate in ADL1 and 3 (*p* = 0.005 and *p* = 0.027, respectively). Changes occurred in the 6MWT with an increase in oxygen uptake (*p* = 0.005) and in the distance covered (*p* < 0.001) and a significant decrease in the Borg scale of dyspnea (*p* = 0.004).

**Conclusion:** Elderly patients with chronic heart failure who are engaged in a short-term residential CR program improve the performance of routine ADLs.

## Introduction

Chronic heart failure (HF) is a disabling condition with a rising prevalence, especially in the elderly (Bui et al., 2011). The clinical hallmark of the disease is a reduced tolerance to physical exercise (Piepoli et al., 2010). Compared to healthy subjects, chronic HF patients exhibit a reduction of peak aerobic capacity and early onset of dyspnea and fatigue (Ponikowski et al., 2016).

In chronic HF, aerobic exercise tolerance usually relates to habitual physical activity level, and progression of HF symptoms is associated with a progressive decrease of patients’ habitual activities (Mezzani et al., 2000).

Elderly chronic HF patients often complain of greater limitations when performing Activities of Daily Living (ADLs; Holland et al., 2010; Dunlay et al., 2015). In particular, older subjects describe a debilitating and distressing condition during daily life situations, due to several detrimental physical and psychosocial issues (Yu et al., 2008).

Spruit et al. (2011) have shown that, differently from healthy elderly subjects, chronic HF elderly patients had an oxygen uptake at a higher proportion of their peak aerobic capacity, and compared to healthy elderly subjects have a higher heart rate and greater ventilation (VE) during simple activities, such as getting dressed, folding towels, or cleaning the floor.

Moreover, Mapelli et al. (2020) recently observed that, in severe chronic HF patients with reduced ejection fraction (HFrEF), the VO_2_ used during ADLs tasks was even higher of peak VO_2_ at cardiopulmonary exercise test (CPET), with a respiratory exchange ratio around or above 1. Moreover, patients were able to compensate for the high percentage of peak VO_2_ needed for a given ADL by prolonging the time needed to complete the task (Mapelli et al., 2020).

Cardiac rehabilitation (CR), which consists in a multidisciplinary and comprehensive treatment including exercise training, drug management, lifestyle modification, and psychosocial counseling, is recommended for the long-term management of HFrEF (Ponikowski et al., 2016). Aerobic exercise training is one component of CR, as its benefit in improving peak exercise capacity, increasing the tolerance to exercise, and reducing the impact of dyspnea and fatigue on the individual’s functional capacity have been widely demonstrated (Taylor et al., 2014).

However, the improvement of exercise tolerance could not always translate into an increased ability to perform functional activities, such as ADLs.

Because the improvement in the ability to perform tasks is an important goal for patients attending CR, more insights are needed on the possible beneficial effects of CR on the performance during ADLs, in terms of symptoms, time spent, and physiological response to the activity.

Single physiotherapy interventions, such as resistance training, can improve the ability of chronic HF patients to perform ADLs (Savage et al., 2011). However, to our knowledge, no studies yet have evaluated the effects of a CR intervention on ADLs performance.

Therefore, the present study aimed to describe the metabolic response during ADLs in elderly HF patients, before and after a residential CR program. Secondary aims were to describe: (a) the change in time necessary to perform five time-related ADLs (b) the modifications of heart rate and symptoms (c) the differences in response between patients in NYHA class 2 and 3 between patients with and without reduced ejection fraction (EF). The hypothesis was that CR might influence metabolic requests during ADL improving efficiency of tasks.

## Materials and Methods

This was a prospective, multicenter, observational study performed at the Cardiac Rehabilitation Department of the Istituti Clinici Scientifici Maugeri IRCCS Network, Italy in the rehabilitative hospitals of Lumezzane (BS), Montescano (PV), Tradate (VA), and Pavia (PV). The study was approved by the local Ethical Committee (CE n. 2204, date 29/05/2018). All participants gave written informed consent to participate in the study.

### Subjects

Diagnosis of chronic HF was made according to the criteria of the latest guidelines of the European Society of Cardiology (Ponikowski et al., 2016).

Patients were included if they were older than 65 years, classified in NYHA class II or III, clinically stable (no need for intravenous therapy), and able to perform a 6-min walking test and to climb 1-floor of stairs. Exclusion criteria were: (a) the presence of cognitive impairment or motor disability; (b) oxygen therapy; (c) recent (within 6 months) cardiac surgery.

### Study Design

All chronic HF patients participated in a comprehensive short-term residential CR program (mean days 19 ± 7). The patients underwent a group-based exercise program, 6 days/week, of low-moderate intensity aerobic training on cycle ergometer or treadmill, supervised by a physiotherapist. The endurance training sessions lasting 40 min/day started from a workload of 0 watts and a day-by-day progression was performed using the Maltais protocol (Maltais et al., 1997) increasing 10 watts for each further step. Patients also performed a circuit group resistance training lasting 20 min/day.

Patients received also nutritional modulation, optimization of drug treatment and education on adherence to medication and on healthy lifestyle, and psychosocial counseling.

### Measures

At baseline, all patients underwent a routine assessment of demographics and anthropometrics data, 2D-echocardiography, and blood chemistry test including NT-proBNP.

### ADL Tests Battery

Before and after CR, participants performed an ADL battery test in the gym.

The metabolic values of ADLs were collected and assessed using a wearable ergospirometer (K5, Cosmed, Rome, Italy; Perez-Suarez et al., 2018) that allows a breath-by-breath measurement of the metabolic and ventilatory response during exercise through a dedicated mask (V2 Mask, Hans Rudolph INC). The device weighs approximately 2 kilograms and is worn through a dedicated backpack. The K5 ergospirometer was calibrated every day following factory instructions. Heart rate (HR) was monitored through a HR monitor placed on the chest through a band (Polar t31, Polar Electro Oy, Kempele, Finland). Collected data were analyzed at a later stage.

Patients were instructed to perform the ADLs at self-selected pace. Between activities, HF patients rested on a chair for at least 4 min, or as long as needed to obtain the return of VO_2_ to baseline level.

Symptoms of dyspnea and fatigue were scored at the beginning and end of each single ADL using a modified Borg symptoms scale (Levinger et al., 2004) ranging from 0 (no symptoms) to 10 points (worst symptoms).

The ADL battery test consisted of six domestic ADLs performed consecutively according to Mapelli et al. (2020) protocol.

The ADLs were:

- ADL1: putting on a pair of socks, a pair of shoes and a vest, sitting on a chair;
- ADL2: folding eight towels, standing;
- ADL3: putting six plastic bottles of water (1.5 L) in a cupboard;
- ADL4: making a bed (standing and walking);
- ADL5: sweeping the 9-m floor dusted with confetti for 4 min (standing and walking);
- ADL6: climbing 1 flight of stairs up and then down carrying a 1.5 kg;
- ADL7: a standard 6-Minute Walking Test (6MWT; The American Thoracic Society, 2002).

For ADL7 (6MWT), HF patients were asked to walk at their own maximal pace along a 35-m long, flat, and straight hospital corridor. The test was symptom-limited and supervised by a physiotherapist. The distance covered during the test was recorded in meters. Cardio-respiratory parameters, Borg scale, and HR were also collected as before.

Two ADLs (5 and 7) had a fixed execution time and non-fixed speed, while the other five ADLs were non-fixed execution time, and the time spent to complete each activity was noted.

TaskVO_2_ [ml/(kg*min)], Task aerobic work (ml), VE/VCO_2_ (ratio), respiratory exchange ratio (RER), and symptoms were measured for each ADL on the last 30 s of exercise. Task aerobic work was stated as the total VO_2_ consumption defined as the Area Under the Curve of O_2_ kinetics. Breath-by-breath data of each test were extracted from an Excel format (Excel, Microsoft Office, Microsoft TM, Redmond, United States).

### Statistics

Data were analyzed by STATA 13.1 software (Stata Corp, LLC). Continuous data were described as mean (standard deviation) and categorical and binary ones as percentage. All statistical tests were applied to overall population and to NYHA class 2 and 3 subgroups.

For all ADLs, we evaluated the mean cumulative value of each variable at baseline and after CR and significant (*p* < 0.05) changes were certified.

For non-fixed execution ADLs, the duration for each activity and the cumulative time were calculated and compared.

For continuous variables, pre-to-post changes in metabolic responses were compared using paired t test and differences between groups were evaluated using unpaired t test. A chi-square test was used to evaluate differences between groups for categorical and binary variables. A value of *p* < 0.05 was considered significant.

## Results

Table 1 shows the baseline demographic, anthropometric, and clinical characteristics of HF patients enrolled in the study. The mean age was 72 ± 5 years. Patients were mainly male (80%), with moderate HF: more than 30% of patients were in NYHA class 3. Mean NT-proBNP was measured in 83 patients and was 2,559 ± 4,511 pg/ml. About 60% of patients had left ventricular EF lower than 40% and two or more comorbidities were present in 60% of patients. Fifty-six percent of patients had an ICD or CRT device implanted according to ESC guidelines recommendations (Ponikowski et al., 2016), while 90% received an angiotensin-converting enzyme inhibitor or one of its alternative and beta-blockers. No major differences in clinical characteristics emerged when patients were grouped according to NYHA functional class 2 or 3 other than a higher prevalence of kidney dysfunction among NYHA class 3 patients.

**Table 1:** Demographic and clinical characteristics of patients enrolled.

Measurement	OVERALL	NYHA 2	NYHA 3	*p* value
	*N* = 99	*N* = 63	*N* = 36	
Age, years	72 ± 5	72 ± 5	73 ± 5	0.322
Sex, male, *N* (%)	79 (79.8)	51 (81.0)	28 (77.8)	0.705
BMI, kg/m^2^	27.0 ± 4.9	26.5 ± 4.6	27.9 ± 5.4	0.177
BMI > 30, *N* (%)	18 (18.2)	10 (15.9)	8 (22.2)	0.431
CIRS, score	2.1 ± 0.9	2.0 ± 0.9	2.1 ± 0.9	0.644
Hemoglobin (Hb), g/dl	13.2 ± 1.6	13.4 ± 1.5	12.8 ± 1.6	0.050
Anemia, *N* (%)	37 (37.7)	39.68 (25.0)	33.33 (12.0)	0.530
NT-proBNP (*N* = 83), pg/ml	2,559 ± 4,511	2,261 ± 4,651	3,084 ± 4,279	0.428
EF, %	38.4 ± 13.4	37.6 ± 13.1	39.8 ± 14.1	0.436
EF < 40%, *N* (%)	60 (60.6)	39 (61.9)	21 (58.3)	0.726
PAP (*N* = 84), mmHg	37.1 ± 13.4	36.5 ± 14.6	38.3 ± 11.0	0.554
TAPSE (*N* = 88), mm	19.1 ± 4.5	19.1 ± 5.1	19.0 ± 3.5	0.952
Active smoke or ex-smokers, *N* (%)	19 (18.8)	11 (17.3)	8 (22.2)	0.492
**Comorbidities, *N* (%)**				
Atrial fibrillation	36 (35.6)	21 (33.3)	15 (41.7)	0.407
Hypertension	62 (62.6)	42 (66.7)	20 (55.6)	0.272
Diabetes	30 (30.3)	18 (28.6)	12 (33.3)	0.620
Dyslipidemia	57 (57.6)	35 (55.6)	22 (61.1)	0.591
COPD	17 (16.8)	11 (17.5)	6 (16.7)	0.920
Kidney failure	30 (29.7)	12 (19.1)	18 (50.0)	0.001
Previous bypass	19 (18.8)	13 (20.6)	6 (16.7)	0.630
Previous PTCA	34 (33.7)	22 (34.9)	12 (33.3)	0.873
**Therapy, *N* (%)**				
β-blockers	89 (89.9)	59 (93.7)	30 (83.3)	0.101
ACE/ARB/Sacubitril/Valsartan	92 (92.9)	59 (93.7)	33 (91.7)	0.711
Mineralocorticoid receptor blockers	53 (53.5)	36 (57.1)	17 (47.2)	0.341
Diuretics	87 (87.9)	56 (88.9)	31 (86.1)	0.684
Carrier of ICD/CRD	56 (55.4)	32 (50.8)	24 (66.7)	0.125

**Legend:** *N, number; NYHA, New York Heart Association; BMI, Body Mass Index; kg, kilogram; CIRS, Cumulative Illness Rating Scale; g, gram: dl, deciliter; Anemia = Hb < 13 g/dl for men and < 12 g/dl for women; NT-ProBNP, N-terminal pro b-type natriuretic peptide; EF, Ejection Fraction; PAP, Pulmonary Artery Pressure; mmHg, millimeters of mercury; TAPSE, Tricuspid Annular Plane Systolic Excursion, mm, millimeters, COPD, Chronic Obstructive Pulmonary Disease; PTCA, Percutaneous transluminal coronary angioplasty; ACE, Angiotensin-converting enzyme inhibitors; ARB, Angiotensin receptor blockers; ICD, Implantable Cardioverter Defibrillator; CRT, Cardiac Resynchronization Device*.

Resting metabolic parameters (VO_2_ = 320 ± 111 ml/min, VCO_2_ = 249 ± 97 ml/min, RER =0.78 ± 0.16, VE = 12.7 ± 3.6 L) did not change after CR.

Table 2 describes metabolic values and dyspnea symptoms at the end of each ADL before and after CR in the overall group and in NYHA class 2 and 3 subgroups.

**Table 2:** TaskVO_2_ and aerobic work, VE/VCO_2_, Respiratory exchange ratio, Borg Scale and exercise parameters before and after rehabilitation.

		OVERALL			NYHA class 2			NYHA class 3		
		Pre	Post	*p* value	Pre	Post	*p* value	Pre	Post	*p* value
**Non-fixed execution and speed**										
ADL1	TaskVO_2,_ ml/(kg*min)	8.4 ± 4.0	8.1 ± 3.1	0.415	8.7 ± 4.6	7.9 ± 3.1	0.109	7.8 ± 2.8	8.4 ± 3.2	0.105
	Task aerobic work, ml	561 ± 358	494 ± 279	0.019	559 ± 327	474 ± 248	0.007	564 ± 413	532 ± 329	0.559
	VE/VCO_2_	46.8 ± 7.9	48.1 ± 8.0	0.064	45.1 ± 6.8	47.2 ± 8.7	0.030	49.8 ± 8.8	49.7 ± 6.7	0.933
	RER	0.8 ± 0.1	0.8 ± 0.1	0.680	0.8 ± 0.1	0.8 ± 0.1	0.916	0.8 ± 0.1	0.8 ± 0.1	0.439
	Borg scale, score	0.8 ± 1.3	0.6 ± 0.9	0.062	0.6 ± 1.0	0.4 ± 0.7	0.128	1.2 ± 1.7	0.8 ± 1.3	0.221
	Maximum HR, bpm	77 ± 14	73 ± 11	0.005	75 ± 13	72 ± 10	0.077	81 ± 14	75 ± 12	0.030
	Duration, s	83 ± 35	75 ± 27	0.002	80 ± 29	72 ± 23	0.001	87 ± 44	82 ± 31	0.273
ADL2	TaskVO_2_, ml/(kg*min)	8.9 ± 3.5	8.9 ± 3.7	0.706	9.1 ± 3.5	8.8 ± 3.6	0.290	8.5 ± 3.4	9.2 ± 4.0	0.105
	Task aerobic work. ml	1,092 ± 823	1,055 ± 771	0.640	1,182 ± 926	1,156 ± 814	0.816	934 ± 583	879 ± 663	0.537
	VE/VCO_2_	45.0 ± 7.0	45.6 ± 6.6	0.293	43.5 ± 6.4	44.3 ± 6.4	0.217	47.6 ± 7.3	47.7 ± 6.3	0.912
	RER	0.8 ± 0.1	0.8 ± 0.1	0.747	0.8 ± 0.1	0.8 ± 0.1	0.658	0.8 ± 0.1	0.8 ± 0.1	1.000
	Borg scale, score	1.0 ± 1.4	0.8 ± 1.4	0.101	0.8 ± 1.2	0.7 ± 1.0	0.077	1.3 ± 1.8	1.1 ± 1.9	0.530
	Maximum HR, bpm	77 ± 15	75 ± 13	0.137	78 ± 16	75 ± 13	0.052	76 ± 12	77 ± 13	0.736
	Duration, s	141 ± 67	132 ± 77	0.038	150 ± 71	142 ± 81	0.137	125 ± 55	115 ± 67	0.150
ADL3	TaskVO_2_, ml/(kg*min)	7.0 ± 2.4	7.3 ± 3.0	0.108	7.1 ± 2.5	7.4 ± 2.9	0.334	6.8 ± 2.4	7.3 ± 3.1	0.164
	Task aerobic work, ml	395 ± 369	335 ± 285	0.183	406 ± 407	302 ± 166	0.052	376 ± 296	393 ± 414	0.828
	VE/VCO_2_	47.5 ± 7.7	47.6 ± 7.3	0.832	45.8 ± 6.6	46.4 ± 7.3	0.393	50.4 ± 8.6	49.7 ± 7.0	0.521
	RER	0.8 ± 0.1	0.8 ± 0.1	0.788	0.8 ± 0.1	0.8 ± 0.1	0.916	0.8 ± 0.1	0.8 ± 0.1	0.513
	Borg scale, score	0.7 ± 1.4	0.7 ± 1.1	0.463	0.7 ± 1.5	0.5 ± 0.8	0.266	0.9 ± 1.4	1.0 ± 1.6	0.520
	Maximum HR, bpm	77 ± 13	75 ± 10	0.027	76 ± 13	74 ± 10	0.062	79 ± 12	77 ± 10	0.242
	Duration, s	50 ± 23	49 ± 22	0.383	50 ± 24	49 ± 23	0.304	50 ± 22	50 ± 20	0.988
ADL4	TaskVO_2_, ml/(kg*min)	8.9 ± 3.5	9.4 ± 4.0	0.045	9.5 ± 3.8	9.8 ± 4.3	0.334	7.8 ± 2.7	8.7 ± 3.3	0.035
	Task aerobic work, ml	680 ± 641	681 ± 513	0.984	753 ± 612	742 ± 518	0.834	557 ± 678	578 ± 496	0.744
	VE/VCO_2_	46.1 ± 7.5	46.0 ± 6.6	0.756	44.4 ± 6.6	44.8 ± 6.7	0.601	49.1 ± 8.2	47.9 ± 6.1	0.238
	RER	0.8 ± 0.1	0.8 ± 0.2	0.901	0.8 ± 0.1	0.8 ± 0.2	0.786	0.8 ± 0.1	0.8 ± 0.1	0.757
	Borg scale, score	0.8 ± 1.2	0.9 ± 1.3	0.748	0.7 ± 0.9	0.7 ± 1.0	0.948	1.1 ± 1.5	1.2 ± 1.8	0.702
	Maximum HR, bpm	78 ± 14	78 ± 12	0.930	79 ± 15	78 ± 12	0.410	77 ± 13	79 ± 11	0.311
	Duration, s	87 ± 50	83 ± 46	0.181	93 ± 50	90 ± 50	0.312	76 ± 48	71 ± 36	0.388
ADL6	TaskVO_2_, ml/(kg*min)	10.5 ± 3.4	10.5 ± 3.3	0.916	10.6 ± 3.4	10.6 ± 3.5	0.872	10.3 ± 3.4	10.2 ± 3.1	0.963
	Task aerobic work, ml	658 ± 597	494 ± 310	0.003	651 ± 661	437 ± 196	0.009	671 ± 470	592 ± 431	0.158
	VE/VCO_2_	45.0 ± 7.3	45.8 ± 7.0	0.099	42.9 ± 6.1	44.1 ± 6.2	0.036	48.7 ± 7.9	48.8 ± 7.4	0.916
	RER	0.8 ± 0.1	0.8 ± 0.1	0.154	0.8 ± 0.1	0.8 ± 0.1	0.061	0.8 ± 0.1	0.8 ± 0.1	1.000
	Borg scale, score	1.9 ± 1.9	1.5 ± 2.0	0.004	1.4 ± 1.4	1.0 ± 1.1	0.004	2.8 ± 2.3	2.4 ± 2.7	0.210
	Maximum HR, bpm	84 ± 15	81 ± 16	0.070	84 ± 16	82 ± 13	0.139	84 ± 15	80 ± 19	0.257
	Duration, s	51 ± 22	48 ± 20	0.122	49 ± 18	47 ± 16	0.285	55 ± 27	50 ± 25	0.259
**Fixed execution and non-fixed speed**										
ADL5	TaskVO_2_, ml/(kg*min)	11.2 ± 3.7	11.4 ± 4.3	0.528	11.5 ± 3.6	11.5 ± 3.8	0.982	10.7 ± 3.8	11.3 ± 5.1	0.386
	Task aerobic work, ml	2,361 ± 883	2,342 ± 747	0.831	2,359 ± 895	2,389 ± 745	0.789	2,364 ± 874	2,262 ± 754	0.452
	VE/VCO_2_	43.7 ± 6.3	43.8 ± 6.2	0.891	42.1 ± 5.3	42.6 ± 5.8	0.336	46.6 ± 7.0	45.9 ± 6.2	0.515
	RER	0.8 ± 0.2	0.8 ± 0.1	0.793	0.8 ± 0.1	0.8 ± 0.1	0.672	0.8 ± 0.2	0.8 ± 0.2	0.510
	Borg scale, score	1.5 ± 1.6	1.1 ± 1.7	0.008	1.2 ± 1.3	0.7 ± 1.0	0.004	2.2 ± 1.9	1.9 ± 2.3	0.320
	Maximum HR, bpm	80 ± 17	78 ± 13	0.194	81 ± 17	78 ± 13	0.078	79 ± 16	80 ± 13	0.848
6MWT	TaskVO_2_, ml/(kg*min)	13.3 ± 3.9	14.1 ± 4.0	0.005	13.6 ± 4.0	14.6 ± 3.9	0.003	12.6 ± 3.6	13.2 ± 4.1	0.338
	Task aerobic work, ml	4,766 ± 1770	5,032 ± 1,523	0.097	4,736 ± 1786	5,181 ± 1,446	0.032	4,819 ± 1766	4,771 ± 1,637	0.850
	VE/VCO_2_	43.7 ± 7.2	44.7 ± 7.3	0.113	42.2 ± 6.7	43.3 ± 7.0	0.139	46.3 ± 7.3	47.1 ± 7.3	0.482
	RER	0.8 ± 1.1	0.9 ± 0.1	0.303	0.9 ± 0.1	0.9 ± 0.1	0.302	0.8 ± 0.1	0.8 ± 0.1	0.751
	Borg scale, score	2.9 ± 2.2	2.3 ± 2.2	0.004	2.5 ± 1.9	1.8 ± 1.6	0.003	3.6 ± 2.6	3.3 ± 2.8	0.381
	Maximum HR, bpm	89 ± 18	90 ± 18	0.184	91 ± 18	92 ± 17	0.533	85 ± 17	88 ± 18	0.208
	Meters	410 ± 90	438 ± 91	<0.001	432 ± 84	461 ± 90	<0.001	372 ± 88	399 ± 80	0.006

**Legend:** *ADLs indicate activities of daily living; ADL1: putting on a pair of socks (sitting on a chair), a pair of shoes (sitting on a chair), and a vest (standing); ADL2: folding 8 towels (standing); ADL3: putting 6 bottles of water (1.5 L) in a cupboard (standing); ADL4: making a bed (standing and walking); ADL5: sweeping a 9-m floor dusted with confetti for 4 min (standing and walking); ADL6: climbing 1 flight of stairs up and then down carrying a 1.5 kg load. 6MWT, 6 minutes walking test; Non-fixed execution and speed: activities with intensity and duration self-adjusted by subjects (ADL1, ADL2, ADL3, ADL4, ADL6.) Fixed execution and non-fixed speed: activities with intensity self-adjusted by subjects but fixed exercise duration (ADL5 and 6MWT); HF, heart failure; HR, heart rate; RER, respiratory exchange ratio; Task VO_2_, total task-related VO_2_; VE/VCO_2_, ventilatory efficiency; VO_2_, oxygen uptake*.

After CR, Task VO_2_ did not change in none of the domestic ADLs, apart in ADL4 (*p* = 0.045) and in 6MWT (*p* = 0.005). Task aerobic work showed a significant reduction in two out of the six domestic ADLs (ADL1 and ADL6, *p* = 0.019 and *p* = 0.003 respectively).

**Figure 1:**
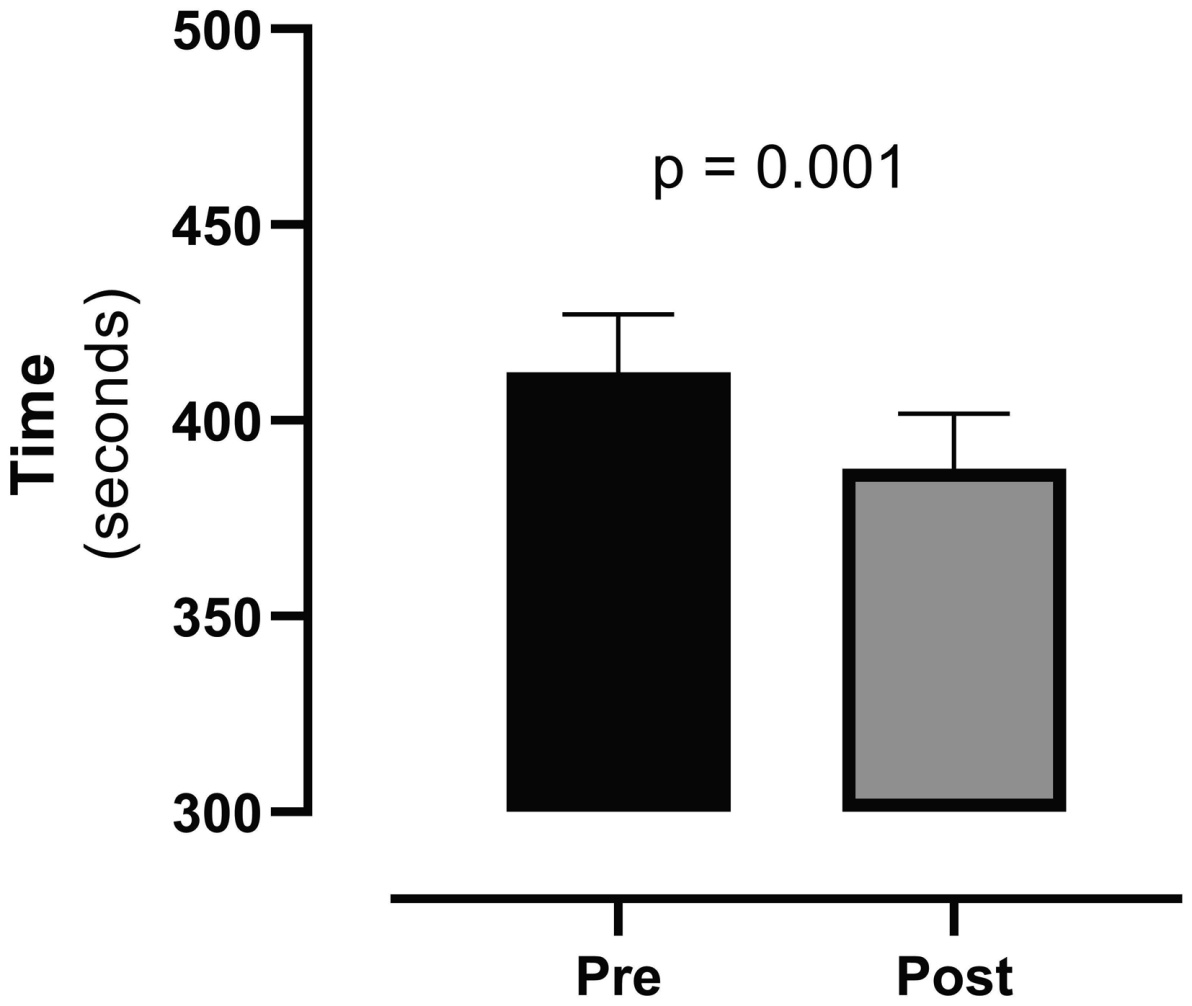
Cumulative time of non-fixed execution and speed ADLs before (pre) and after (post) cardiac rehabilitation. The boxes refer to mean and bars refer to standard error mean (SEM).

Notably, for non-fixed execution and speed ADLs, there was a decrease in the cumulative time required to perform the task-related activities (ADL 1–4 and ADL6; from 412 ± 147 to 388 ± 141 s, *p* = 0.001, Figure 1). Moreover, we found a pre-to-post reduction in maximal HR during ADL1 and ADL3 (p = 0.005 and *p* = 0.027, respectively) and a reduction of dyspnea during ADL6 (*p* = 0.004).

For the fixed execution and non-fixed speed activities (ADL5 and 6MWT), we observed a relevant increase in the distance covered at the 6MWT (*p* < 0.001) and a significant decrease in the discomfort of breathing in both activities (Borg scale of dyspnea ADL5, *p* = 0.008 and Borg scale of dyspnea of 6MWT, *p* = 0.004).

We also explored the relationship between NT-proBNP and the improvement in outcome measures of CR (change in meters and VO_2_ at 6MWT and cumulative time of ADL). A weak, albeit statistically significant correlation, was found only between NT-proBNP and change in 6MWT distance (*R* = 0.2305, *p* = 0.036).

**Figure 2:**
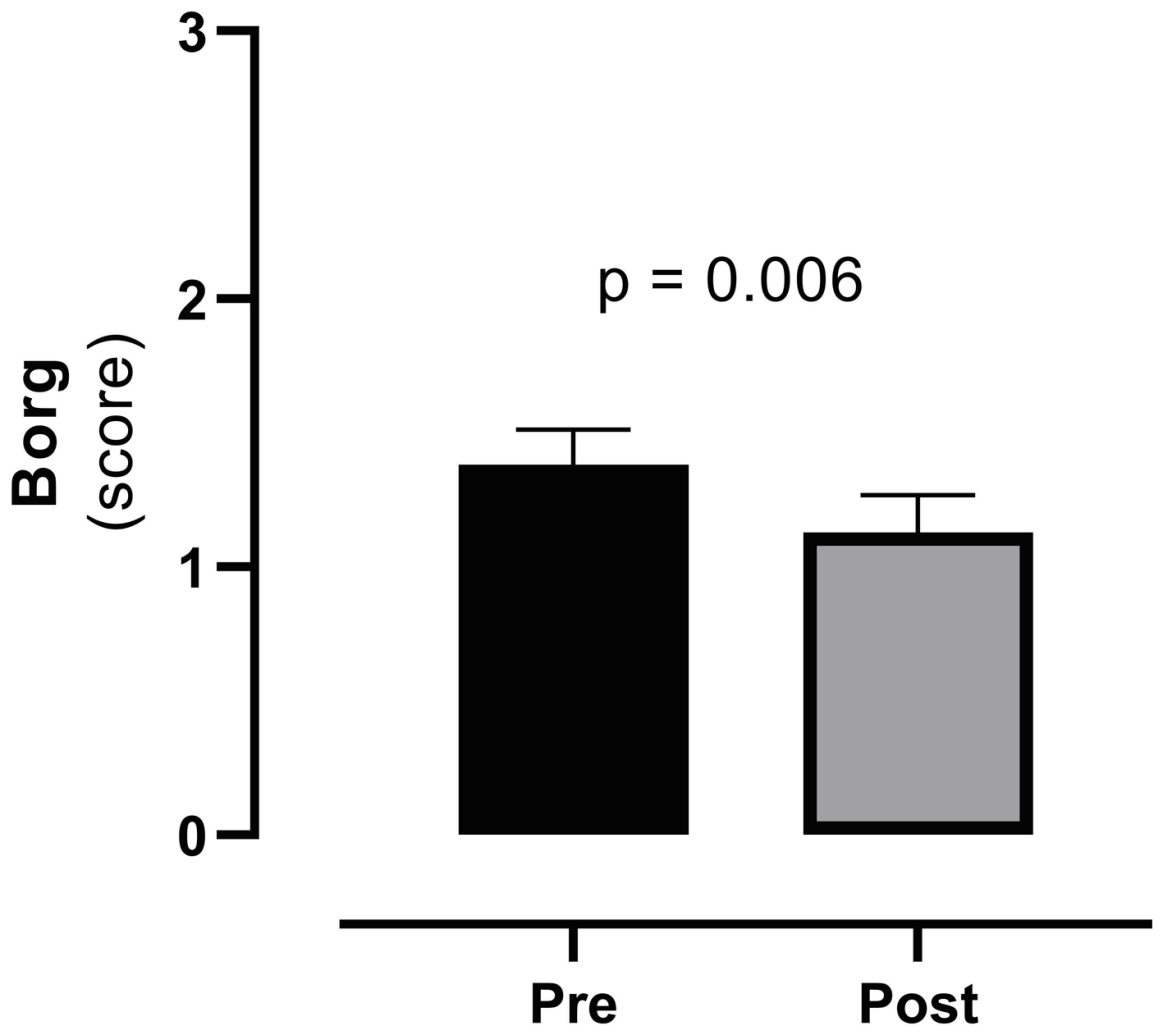
Cumulative value of the Borg scale before (pre) and after (post) cardiac rehabilitation. Legend: the boxes refer to mean and bars refer to SEM.

Finally, as the complaint of dyspnea seriously compromises the ability of patients to perform ADLs, we compared the mean cumulative value of the Borg scale before and after CR and found an overall symptom reduction (from 1.38 ± 1.31 to 1.13 ± 1.38; *p* = 0.006, Figure 2).

### Differences Between NYHA Class 2 and 3

At baseline, as far as the domestic ADLs, there were no differences between NYHA class 2 and NYHA class 3 patients in all parameter, but the VE/VCO_2_ ratio was significantly higher in NYHA class 3 patients (*p* < 0.05 for each ADL). A further difference was related to HR that was higher in NYHA 3 patients during the execution of ADL1 (*p* = 0.023). As far as the baseline 6MWT (ADL7), patients in NYHA class 3 besides the higher VE/VCO_2_ ratio covered a significantly lower distance with respect to NYHA 2 patients (*p* = 0.001).

After residential CR, variations in metabolic and cardio-respiratory parameters were more evident in NYHA class 2 patients where changes were present for the Task aerobic work in ADL1, ADL6 and 6MWT (*p* = 0.007, *p* = 0.009 and *p* = 0.032, respectively), duration in ADL1 (*p* = 0.001) and Borg scale of dyspnea in ADL5, ADL6 (*p* = 0.004 for both).

NYHA class 2 patients also had improvements in the performance of the 6MWT with a significant increase in Task VO_2_ (*p* = 0.003) and in the distance walked (*p* < 0.001) and a significant reduction in the feeling of dyspnea (*p* = 0.003). By contrast, in NYHA class 3 patients after CR, only the distance walked at the 6MWT was found to be increased (*p* = 0.006).

### Differences Between Patients With Normal/Reduced Ejection Fraction

No differences between baseline metabolic and cardio-respiratory variables during all ADLs in patients with and without EF < 40% were found.

At the end of CR treatment, rates of angiotensin-converting enzyme inhibitors or one of their alternatives and mineral corticoid receptor blockers were increased to 94.5% and to 55.6%, respectively.

## Discussion

To our knowledge, this is the first study showing that, in elderly chronic HF patients, a short-term residential CR program improves the capacity to perform ADLs.

We extended our analysis to HF severity (NYHA class) and to those patients with reduced and preserved EF; NYHA 2 elderly HF patients were more sensible to CR program, even though 6MWT was improved in both subgroups (i.e., NYHA class 2 and 3), while HF elderly patients with reduced EF and those with preserved EF had a comparable behaviour in cardiometabolic response after CR.

As advancing age accelerates the functional impairment that characterizes HF, many elderly chronic HF patients also experience a decline in the ADLs, which contributes to loss of independence and worsens quality of life (Dunlay et al., 2015). Although the ability to perform ADLs is an important patient-related outcome, very few studies focused at their improvement. Gary et al. (2011) assessed the effects of a 12-week home-based exercise program on the time required for dressing, kitchen, household activities, and endurance walking in a sample of 24 patients with NYHA class 2 or 3 systolic HF between the ages of 40 and 75 years. The participants in the exercise group improved their time in seconds compared to baseline for completing most tasks and increased weight carried, while there were no changes in the control group. In the recent REHAB-HF trial, a progressive rehabilitation intervention initiated during hospitalization for HF and continued for 36 outpatient sessions improved physical function domains as assessed by the Short Physical Performance Battery (Kitzman et al., 2021).

At variance with the quoted studies, duration of intervention in our study did not exceed 25 sessions (mean length of stay 19 days), but is it worth to underscore that exercise was just one of the components of our comprehensive rehabilitation program.

A relevant question is whether these improvements had to be ascribed to the exercise training alone or whether other components of the rehabilitation intervention also concurred to the observed benefits. Theoretically, according to literature data showing an improvement in functional capacity and cardiopulmonary parameters in patients with HF (Giallauria et al., 2020; Malfatto et al., 2020; Dos Santos et al., 2021), we cannot dismiss the possibility that the optimization of pharmacological treatment might also have played a role. However, we have to underscore that, in our study, changes in therapy concerned a very limited number of patients over a limited number of days.

### Metabolic Requirement of ADLs

Few studies addressed the metabolic requirement of ADLs in patients with HF. Spruit et al. (2011) analyzed data from CPET and the performance of 5 simple domestic ADLs in 23 stable chronic HF patients (45–80 years of age) who were compared to 20 healthy peers. Task-related oxygen uptake was similar in the majority of ADLs between chronic HF patients and healthy subjects. However, patients with chronic HF performed ADLs at a higher proportion of their peak oxygen uptake than healthy peers. In this study (Spruit et al., 2011), sweeping the floor for 4 min (patients with chronic HF used 52% of the peak aerobic capacity assessed during CPET) resulted in the higher task-related oxygen uptake. Moreover, for ADLs not requiring a fixed execution time, chronic HF patients needed more time to complete them (Spruit et al., 2011). Mapelli et al. (2020) studied 60 patients with HF and 40 healthy volunteers and confirmed the importance of prolongation of exercise time during ADLs with no fixed duration, as a means to reduce metabolic cost and optimize effort. Notably, patients with severe HF had a greater prolongation of exercise with respect to patients with less severe HF (Mapelli et al., 2020).

Our data cannot compare with the above studies, as we did not enroll healthy controls, there are differences in the clinical phenotypes of HF (our study included also HF patients with reduced and preserved EF), and most importantly, the aim of our study was to assess changes in the metabolic requirement of ADLs following a comprehensive CR program.

### Effects of Cardiac Rehabilitation on the Metabolic Requirements of ADLs

Following our short-term comprehensive CR program, we did not observe significant changes in the TaskVO_2_ of any of the ADLs, although there was a reduction in Task aerobic work in ADL1 and ADL6.

However, there was a significant reduction in the cumulative time required to perform those activities characterized by a non-fixed duration (ADL 1–4 and ADL6, Figure 1) and a reduction in maximal heart rate in ADL1 and ADL3. This is well in line with the already quoted findings from Gary (Gary et al., 2011) and supports the hypothesis of Mapelli that, to perform a given exercise, patients with HF spontaneously adapt to the task by self-regulating the effort intensity through a time prolongation (Mapelli et al., 2020). Indeed, CR, by improving physical function and muscle deconditioning ended up in a reduction of the time needed to perform those ADLs like getting dressed, folding towels, or making a bed for which there was no limit in time duration.

The 6MWT is the most widely used test to measure functional capacity of HF patients in the CR setting. Peak aerobic capacity increased during 6WMT after CR, along with an increase in the distance walked and a reduction in the Borg symptom scores for dyspnea.

Benefits of CR were more evident in NYHA class 2 patients. However, fewer patients were categorized as NYHA class 3, thus raising the possibility that the limited number of patients might have prevented from reaching statistical significance in some differences. Indeed, the extent of change in the distance covered at 6MWT (NYHA 2 = 29.0 ± 41.0 meters vs. NYHA 3 = 26.8 ± 55.1 meters, *p* = 0.820) and the duration of non-fixed execution and speed ADLs (NYHA 2 = − 23.6 ± 57.6 s vs. NYHA 3 = − 25.7 ± 96.1 s, *p* = 0.840) were similar between class 2 and 3 patients. Thus, we can assume that benefits of CR were also present in NYHA class 3 patients.

### Limitations of the Study

The study has some limitations that should be addressed and that limit generalization of the results. First, the lack of a control group of healthy subjects. Although it was beyond of the scope of our study to address differences in the performance of ADLs between chronic HF patients and healthy subjects, the presence of a control group would have allowed a comparison. Second, the duration of the rehabilitation program. We have restricted our intervention to the residential phase of CR. Formal rehabilitation programs imply 12-week duration or a minimum of 36 sessions. We cannot exclude that better results might be obtained by prolonging rehabilitation on an outpatient or on a home-based basis. Third, the intensity of the physical training. As a logical consequence of the short duration of the program, intensity of activities remained low to moderate. Finally, a control group of HF patients who did not perform the CR program is lacking. Indeed, we cannot exclude a learning effect on ADLs.

## Conclusion

Elderly chronic heart failure who are engaged in a short-term residential cardiac rehabilitation program improve the performance of routine ADLs.

There is a need for further studies to identify and evaluate the efficacy of innovative treatments targeting improvement in ADLs as a strategy to improve clinical and patient-reported outcomes in elderly patients with chronic HF.

## Data Availability Statement

Anonimyzed data and materials will be made publicly available at: https://www.zenodo.org.

## Ethics Statement

The studies involving human participants were reviewed and approved by ICS Maugeri Ethical Committee (EC n. 2204, date 29/05/2018). The patients/participants provided their written informed consent to participate in this study.

## Author Contributions

MTLR, SS, and PA contributed to the conception and design of research. MP, RR, AM, and ML conducted the experimental sessions. RM performed the statistical analysis. MP, MTLR, SS, and UC interpreted the results. MP and MTLR drafted the manuscript. MP, SS, UC, PA, and MTLR edited and revised critically the manuscript. All authors contributed to the article and approved the submitted version.

## Funding

This work was supported by the “Ricerca Corrente” Funding scheme of the Ministry of Health, Italy.

## Conflict of Interest

The authors declare that the research was conducted in the absence of any commercial or financial relationships that could be construed as a potential conflict of interest.

## Publisher’s Note

All claims expressed in this article are solely those of the authors and do not necessarily represent those of their affiliated organizations, or those of the publisher, the editors and the reviewers. Any product that may be evaluated in this article, or claim that may be made by its manufacturer, is not guaranteed or endorsed by the publisher.
